# Surfactant Protein D as a Potential Biomarker and Therapeutic Target in Ovarian Cancer

**DOI:** 10.3389/fonc.2019.00542

**Published:** 2019-07-09

**Authors:** Juhi Kumar, Valamarthy Murugaiah, Georgios Sotiriadis, Anuvinder Kaur, Jeyarooban Jeyaneethi, Isotta Sturniolo, Fatimah S. Alhamlan, Jayanta Chatterjee, Marcia Hall, Uday Kishore, Emmanouil Karteris

**Affiliations:** ^1^Department of Life Sciences, College of Health and Life Sciences, Brunel University London, Uxbridge, United Kingdom; ^2^Department of Infection and Immunity, King Faisal Specialist Hospital and Research Centre, Riyadh, Saudi Arabia; ^3^Faculty of Health and Medical Sciences, School of Biosciences and Medicine, University of Surrey, Guildford, United Kingdom; ^4^Mount Vernon Cancer Centre, Northwood, United Kingdom; ^5^Institute of Environment, Health and Societies, Brunel University London, Uxbridge, United Kingdom

**Keywords:** surfactant protein, SP-D, innate immunity, ovarian cancer, biomarker, circulating tumor cells

## Abstract

Surfactant protein D (SP-D) is an important innate immune molecule that is involved in clearing pathogens and regulating inflammation at pulmonary as well as extra-pulmonary sites. Recent studies have established the role of SP-D as an innate immune surveillance molecule against lung and pancreatic cancer, but little is known about its involvement in signaling pathways it can potentially activate in ovarian cancer. We focused our study on ovarian cancer by performing bioinformatics analysis (Oncomine) of datasets and survival analysis (Kaplan-Meier plotter), followed by immunohistochemistry using ovarian cancer tissue microarrays. SP-D mRNA was found to be expressed widely in different types of ovarian cancer irrespective of stage or grade. These *in silico* data were further validated by immunohistochemistry of clinical tissues. High transcriptional levels of SP-D were associated with unfavorable prognosis (overall and progression-free survival). We also detected SP-D protein in Circulating Tumor Cells of three ovarian cancer patients, suggesting that SP-D can also be used as a potential biomarker. Previous studies have shown that a recombinant fragment of human SP-D (rfhSP-D) induced apoptosis in pancreatic cancer cells via Fas-mediated pathway. In this study, we report that treatment of SKOV3 cells (an ovarian cancer cell line) with rfhSP-D led to a decrease in cell motility and cell proliferation. This was followed by an inhibition of the mTOR pathway activity, increase in caspase 3 cleavage, and induction of pro-apoptotic genes Fas and TNF-α. These data, suggesting a likely protective role of rfhSP-D against ovarian cancer, together with the observation that the ovarian cancer microenvironment overexperesses SP-D leading to poor prognosis, seems to suggest that the tumor microenvironment components manipulate the protective effect of SP-D *in vivo*.

## Introduction

Surfactant protein D (SP-D) is an innate immune molecule involved in clearing a range of pathogens and apoptotic/necrotic cells at pulmonary as well as extra-pulmonary mucosal sites (1, 2). SP-D interacts with infectious microbes using its C-type lectic domains and brings about effector mechanisms via agglutination, enhanced phagocytosis and oxidative burst, and direct cytostatic properties (1); SP-D is also considered to be involved in controlling allergic sensitization in the lungs (3) where it exerts anti-lymphoproliferative effect following allergen challenge (4). In mouse models of Aspergillus fumigatus-induced allergic hypersensivity, immunopathological parameters of type I and III hypersensitivity improved considerably following therapeutic delivery of full-length human SP-D and its recombinant fragment containing trimeric neck and lectin domains (rfhSP-D) (5). SP-D can also induce apoptosis in activated eosinophilic and T cells via p53 pathway (6–9). In addition to several extrapulmonary sites, SP-D has also been found in various parts of the reproductive tract, including the ovaries (2, 10).

Emerging evidence seems to suggest that tumor cells are affected by the inflammatory environment, which can influence tumor proliferation and metastasis (11). Recent studies have focused on lung cancer, as it is the primary production site of SP-D where it plays a crucial role in surfactant and immunological homeostasis. SP-D can downregulate the EGF pathway by directly binding to EGFR and inhibit cell proliferation, invasion and metastasis of the A549 lung cancer cell line (12). A clinical study in 71 patients showed that low levels of SP-D were associated with increased incidence of lung cancer and that SP-D levels in bronchoalveolar lavage fluid could be used as a biomarker (13). Moreover, single nucleotide polymorphisms within the SP-D gene have been correlated with lung cancer, pneumonia and emphysema (14, 15). In pancreatic cancer, a recombinant fragment of human SP-D (rfhSP-D) induced apoptosis *in vitro* via Fas-mediated pathway (16). Furthermore, rfhSP-D inhibited TGF-β expression in a range of pancreatic cancer cell lines, thereby reducing their invasive potential by suppressing the Epithelial-to-Mesenchymal Transition (17).

In a recent study, it has been demonstrated that SP-D mRNA is overexpressed in ovarian cancer and can be of a potential prognostic value (18). Ovarian cancer affects over 65,500 women every year in Europe alone, and within 5 years, nearly 70% (42,716) of sufferers are predicted to die; it is the 6th leading cause of death in women worldwide. Ovarian cancer is known as the silent killer as it causes no symptoms in the early stages, so it is difficult to detect. Even at its advanced stage, the symptoms are very vague. Approximately, 70% of patients are diagnosed at advanced stage III (where ovarian cancer has spread into the upper part of the abdomen), or IV (where ovarian cancer cells are outside the abdominal cavity), with nearly 85% expected mortality. If ovarian cancer is diagnosed/detected early (Stage I–in the ovary only), its treatment is much more successful with the survival rates up to 90% (19, 20). In this study, we carried out a bioinformatic analysis to ascertain the potential prognostic role of SP-D in ovarian cancer. We also used a tissue microarray to validate these data and circulating tumor cells from ovarian cancer patients to measure/detect SP-D levels in liquid biopsies.

Given its pro-apoptotic and anti-invasive potential in a few *in vitro* cancer models, we hypothesized that rfhSP-D might be a novel therapeutic agent in ovarian cancer. Thus, in this study, we also investigated the effect of rfhSP-D on ovarian cancer signaling, using the SKOV3 cell line as an *in vitro* model- with emphasis to mTOR pathways. The mTOR pathway is a central regulator of growth, proliferation, apoptosis and angiogenesis providing balance between cellular resources. mTOR and one of its substrates, S6 kinase, are activated in ovarian cancer cells and inhibition of the mTOR pathways has anti-proliferative effects (21).

## Materials and Methods

### *In silico* Analyses

The expression level of SFTPD gene in various types of ovarian cancer was analyzed using Oncomine, a cancer microarray database and web-based data mining platform from genome-wide expression analyses (22, 23). We compared the differences in mRNA level between normal and ovarian cancer tissues. The prognostic significance of SP-D mRNA expression and survival in ovarian cancer was analyzed by Kaplan–Meier plotter as described recently (18).

### Immunohistochemistry

Ovarian carcinoma tissue microarrays, containing 10 cases of ovarian tumor with 2 non-neoplastic tissues, duplicated cores per case (Biomax, U.S.), were used to examine the expression of SP-D. The slides were deparaffinised following a series of washing in histoclear (National Diagnostics) and ethanol. Slides were boiled in sodium citrate for 20 min, washed with Tris-buffered Saline (TBS) with 0.025% v/v Triton-X 100, and then incubated with 3% v/v hydrogen peroxide in PBS for 30 min before being washed again in TBS with 0.025% Triton X 100. After blocking the slides using 5% v/v goat serum in PBS + 0.025% Triton-X 100 in a humidity chamber, the slides were incubated overnight with rabbit anti-human SP-D polyclonal antibodies (MRC immunochemistry unit, Oxford) diluted in PBS + 0.025% Triton-X 100. After a series of washing with TBS + 0.025% Triton X 100, the slides were incubated with Goat anti-rabbit IgG-HRP conjugate (Zytomed Systems, Germany) diluted in PBST, for 1 h at room temperature. DAB (3,3′-diaminobenzidine) substrate solution (Vector Laboratories) containing hydrogen peroxide was loaded on the slides for 5 min. Slides were washed in H_2_O for 5 min and then incubated with Harris' haematoxylin for 30 s. Slides were stained with 0.1% w/v sodium bicarbonate for 30 s before dehydration in ethanol and histoclear. Images were captured using an EOS 1200D Brunel Microscope Ltd.

### Liquid Biopsies

Patients with advanced ovarian cancer included in this study were enrolled at Mount Vernon Cancer Centre (East and North Hertfordshire NHS Trust) in the prospective CICATRIx clinical study that allows collection of blood samples for biomarker studies. All patients provided written informed consent for participation in the study and subsequent use of their tissue and blood specimens for research. The CICATRIx study was approved by the West Midlands—South Birmingham Ethics Committee (reference 16/WM/0196).

### ImageStreamx Mark II Flow Cytometry

Whole blood (1 ml) from ovarian cancer patient samples (*n* = 3) was mixed with 9 ml of red blood cell lysis (RBC) buffer (G Biosciences, St. Louis, MO, USA), followed by incubation for 10 min at room temperature with gentle agitation. Following centrifugation at 1,260 × g for 10 min at 4°C, the supernatant was aspirated and 3 ml of RBC lysis buffer for second wash. This was followed by another incubation for 10 min at room temperature with gentle agitation, followed by centrifugation. The resultant pellet was resuspended in 1.5 ml of PBS and then centrifuged at 1,450 × g for 3 min. The cell pellet was resuspended in 1 ml of ice-cold 4% v/v paraformaldehyde for 7 min on ice and then centrifuged for 5 min at 250 × g. Subsequent immunostaining using specific polyclonal antibodies against human SP-D diluted in FBS-PBS (1:200) was used as previously described in the solution containing CTCs (and some white blood cells) (24, 25).

### Cell Culture and Treatments

SKOV3 (human ovarian clear cell adenocarcinoma) cell line was purchased from the American Type Culture Collection (ATCC, Rockville, MD, USA) and used as an *in vitro* cell model for epithelial ovarian cancer. SKOV3 cells were cultured in DMEM supplemented with 10% v/v FBS, 1% v/v penicillin/streptomycin and 1% L-glutamine (Gibco, MA, USA). Cells were grown under standard culture conditions (37°C, 5% v/v CO_2_). At approximately 80–90% confluency, the media was discarded, and cells were washed with fresh PBS. Seeded SKOV3 cells in 6-well plates were treated with rfhSP-D in a dose- (5, 10, 20 μg/ml) and time- (12, 24, 48 h) dependent manner in triplicates.

### Expression of a Recombinant Fragment of Human SP-D (rfhSP-D)

A recombinant fragment of human SP-D containing trimeric neck and C-type lectin domains was expressed in *E.coli* and purified, as previously described (26, 27). Its three-dimensional crystallographic structures are well-established (28, 29). The affinity purified fractions were passed through a Pierce High Capacity endotoxin removal resin column to remove lipopolysaccharides (LPS) (Thermo Scientific). The endotoxin levels were measured using a QCL-1000 Limulus amebocyte lysate system (BioWhittaker, Walkersville, MD, USA) and were approximately 5 pg per μg of rfhSP-D.

### Immunofluorescence Microscopy for SP-D

SKOV3 cells were grown in a 24-well plate on the top of coverslips. Cells were fixed with 4% paraformaldehyde (PFA; Sigma-Aldrich) for 10 min at room temperature, and then permeabilised with 0.5% v/v Triton X 100 (Sigma-Aldrich) for 5 min on ice. Next, cells were blocked using 5% v/v FBS diluted in PBS for 45 min and then incubated for another 45 min with rabbit anti-human SP-D polyclonal antibody diluted in 5% FBS-PBS. Cells were washed and incubated with a staining buffer containing Alexafluor 568 secondary conjugated goat anti-rabbit antibody, phalloidin 488 and Hoechst (Invitrogen, Life Technologies). This incubation was performed in the dark for 45 min. After washing with 5% FBS-PBS, the coverslips were mounted on slides and visualized under a HF14 Leica DM4000 microscope.

### Live Cell Imaging

SKOV3 cells were grown in a Petri dish and allowed to reach ~30–40% confluency. Cells were treated with rfhSP-D at a final concentration of 10 μg/ml. Cells were then placed under a Zeiss Axiovert 200M microscope attached to an incubator in order to examine cell survival and visualize cell motility. Images of the cells were recorded every 5 min for 15 h. X and Y coordinates were generated through ImageJ software for 25 cells for the first 8 h. Using Pythagoras' theorem, the distance, the velocity and the displacement were established.

### Wound Healing Assay

SKOV3 cells were assessed for their ability to close an artificially created gap in a cell growth area in the presence or absence of rfhSP-D. SKOV3 cells were cultured in a petri dish and allowed to reach 100% confluency. Using a 20 μl pipette tip, the bottom of the dish was scratched to create an artificial gap. Cells were placed under a Zeiss Axiovert 200M microscope and images were recorded every 5 min for up to 48 h.

### Flow Cytometry

SKOV3 (0.1 × 107) cells were grown in a 6-well plate and incubated with rfhSP-D (5, and 10 μg/ml), along with an untreated control, for 48 h. The cells were then detached using 2 × Trypsin-EDTA (0.5%) (Fisher Scientific) and centrifuged at 1,500 × g for 5 min. Annexin V apoptosis detection kit with PI (Abcam) was used, according to the manufacturer's instructions. After extensive washing with PBS, apoptosis was measured using Novocyte Flow Cytometer. Compensation parameters were acquired using unstained, untreated FITC stained, and untreated PI stained. 12,000 SKOV3 cells were acquired for each experiment and compensated before plotting the acquired data.

### Fluorescence Microscopy

SKOV3 cells were cultured in DMEM-F12 medium, supplemented with 10% v/v FBS, penicillin (100 U/ml)/streptomycin (100 μg/ml), and 2 mM L-glutamine (Fisher Scientific). Cells were grown at 37°C in the presence of 5% v/v CO_2_ until 85% confluency was reached. For apoptosis analysis, 0.5 × 10^5^ cells were grown on microscopy coverslips, and incubated with rfhSP-D (10 μg/ml) in serum free medium for 48 h. The cells were then washed with PBS three times, and incubated with FITC Annexin V (1:200), and PI (1:200) diluted in Annexin V-binding buffer, for 15 min at room temperature in dark. After washing again with PBS, the coverslips were mounted on the slides, and visualized under a HF14 Leica DM4000 microscope. For mTOR analysis, the cells were incubated with rfhSP-D (10 μg/ml) for 15 h, and washed gently with PBS three times. The cells were permeablized and fixed with ice-cold 100% methanol for 10 min at −20°C. Following 1 h incubation with rabbit anti-human mTOR (1:500) (Sigma-Aldrich) primary antibody, the cells were washed again with PBS, and incubated with Hoechst (1:10,000) and Goat anti-rabbit Alexa Fluor® 488 (1:500) (Abcam) secondary antibody. The mounted coverslips were visualized on a HF14 Leica DM4000 microscope.

### RNA Extraction and Quantitative RT-PCR

RNA was extracted from SKOV3 cells treated and untreated with rfhSP-D using the Nucleospin kit (Macherey-nagel, Bethlehem, USA) and the quantity was estimated using nanodrop. Two microgram of total RNA was used to synthesize cDNA using the Precision nanoScript 2 Reverse Transcription kit (Primer design). Relative expression levels of mTOR, DEPTOR, Rictor and Raptor (19) were assessed by quantitative PCR (Q-PCR) on an ABI FAST HT9000 PCR instrument, using SYBR green mastermix (Primer Design). Gene expression levels were normalized to GAPDH levels. All samples were analyzed in triplicates (19). RT-qPCR data was analyzed using the ΔCq method whereby the Cq of the endogenous control was subtracted from the Cq of the gene of interest and an RQ (relative quantity) value was calculated by finding 2-ΔCq (30).

### Western Blotting

Proteins were extracted from SKOV3 cells treated with rfhSP-D for 24 and 48 h (21) and separated on a 10% v/v SDS-PAGE. The separated proteins were then electrophoretically transferred onto a nitrocellulose membrane (Thermo Scientific) in Wet-Transfer Buffer. The membrane was incubated in TBS containing 5% w/v dried milk powder and 0.1% v/v Tween Tween-20, for 1 h at room temperature to block non-specific binding. The membrane was then incubated with primary anti-human SP-D polyclonal antibodies (raised in rabbit), caspases 3 and 9, and phospho (Thr389) p70S6K (Cell Signaling Technology) at 4°C overnight. The membranes were washed in TBS + 0.1% Tween-20 (3 times, 15 min each) before incubation with the secondary goat anti-rabbit IgG-HRP-conjugated antibody for 1 h at room temperature. Proteins were visualized as previously described (21).

### Statistical Analysis

Graphpad Prism 5.0 was used to generate the graphs and perform the statistical analysis. When data were of equal variance, an unpaired student's *t*-test was performed. When the data was of unequal variance, the Mann-Whitney U test was performed. Values were considered significant when ^*^p < 0.05, ^*^p < 0.01 and ^***^p < 0.001. Significance was identified between control and treated samples, as well as between treated samples. Error bars represent the standard deviation or standard error of the mean, where specified. Survival curves were generated by the Kaplan–Meier plots. All results are displayed with *p*-values from a logrank test. *p* < 0.05 were considered significant. Similarly, with Oncomine, the statistical significance of data (p-values) was provided by the program.

## Results

### SP-D Is Expressed in Ovarian Cancer and Predicts Survival

A recent study using a bioinformatic approach and RT-qPCR validation indicated that SP-D is overexpressed in ovarian cancer patients (serous cystadenocarcinoma) compared to healthy controls (18). In the current study, we performed an extended bioinformatics analysis in order to investigate whether SP-D is differentially expressed in various types of ovarian cancer and whether it can serve as a potential prognostic marker for the disease. We used the Oncomine™ dataset and the survival analysis platforms Kaplan–Meier plotter.

**Figure 1:**
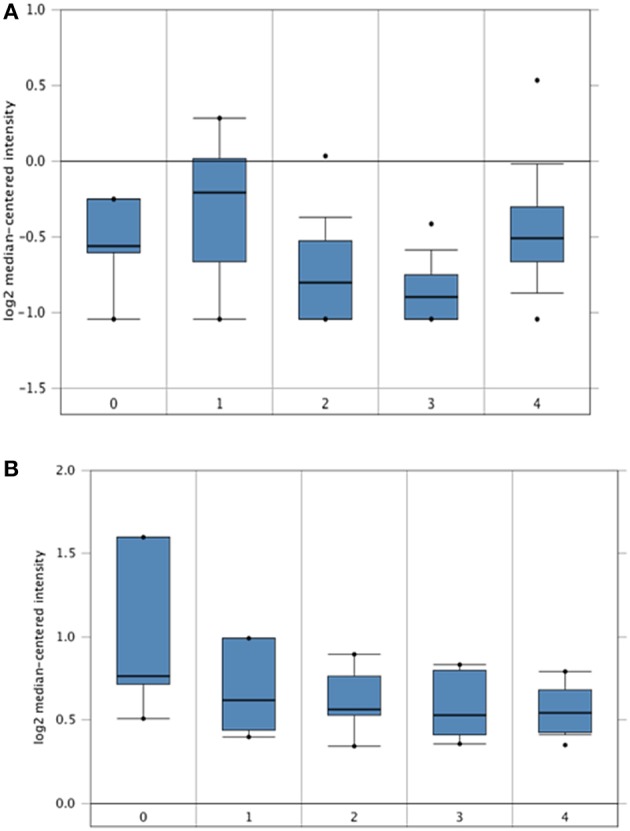
*In silico* analyses of SP-D (SFTPD) expression in different subsets of ovarian cancer patients. SP-D gene expression from the Hendrix et al. (31) dataset plotted by Oncomine. **(A)** Mean SP-D gene expression in control normal ovaries (0), ovarian clear cell adenocarcinoma (1), ovarian endometrioid adenocarcinoma (2), ovarian mucinous adenocarcinoma (3), and ovarian serous adenocarcinoma (4). Boxes represent the 25th-75th percentile (with median line), bars show the 10th−90th percentile and dots show the complete spread of data. Fold change in SP-D expression was 1: 1.217 (*p* = 0.123), 2: −1.109 (*p* = 0.782), 3: −1.201 (*p* = 0.897) and 4: 1.086 (*p* = 0.266) when compared to normal, respectively. SP-D gene expression from the Lu et al. dataset **(B)**. Mean SP-D gene expression in control normal ovaries (0), ovarian clear cell adenocarcinoma (1), ovarian endometrioid adenocarcinoma (2), ovarian mucinous adenocarcinoma (3), and ovarian serous adenocarcinoma (4). Fold change in SP-D expression was 1: −1.179 (*p* = 0.855), 2: −1.214 (*p* = 0.893), 3: −1.255 (*p* = 0.922) and 4: −1.257 (*p* = 0.924) when compared to normal, respectively.

The Hendrix dataset (31) demonstrated that SP-D (SFTPD) is expressed in a wide range of ovarian cancers such as: clear cell adenocarcinoma (*n* = 8), endometrioid adenocarcinoma (*n* = 37), mucinous adenocarcinoma (*n* = 13), serous adenocarcinoma (*n* = 41) and normal ovaries (*n* = 4). Some subtle changes were noted amongst these groups, but they did not reach significance compared to healthy controls (Figure 1A). Similarly, when the Lu dataset was accessed (32), a similar picture emerged with the expression of SP-D mRNA (SFTPD) in normal ovaries (*n* = 5), clear cell adenocarcinoma (*n* = 7), endometrioid adenocarcinoma (*n* = 9), mucinous adenocarcinoma (*n* = 9) and serous adenocarcinoma (*n* = 20; Figure 1B).

**Figure 2:**
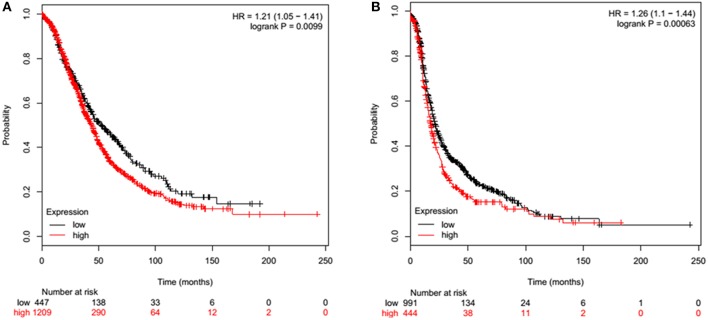
Overall Survival (OS) of ovarian cancer patients expressing high (red) and low (black) SP-D **(A)**. High (*n* = 1209) and low (*n* = 447) expression of SP-D is shown over a 250 month (20 year) period. Low SP-D expression shows a significant improvement in survival than high SP-D expression (*p* = 0.0099). Progression-Free Survival (PFS) of ovarian cancer patients expressing high (red) and low (black) SP-D **(B)**. High (*n* = 444) and low (*n* = 991) expression of SP-D is shown over a 250 month (20 year) period. There is greater overall survival in low SP-D expression compared to high SP-D expression (*p* = 0.00063).

Overall Survival (OS) in ovarian cancer was measured by Kaplan-Meier plots. In a cohort of 1656 ovarian cancer patients, high (*n* = 1209) and low (*n* = 447) mRNA expression of SP-D was measured over a 250-month (20 year) period. Low SP-D expression shows a significant improvement in survival than high SP-D expression (*p* = 0.0099; Figure 2A). Similarly, Progression-Free Survival (PFS) was also studied in 1435 ovarian cancer patients of which 444 had high expression of SP-D and 991 low. There was a significant progression-free survival (PFS) improvement in low SP-D cohort compared to patients with high SP-D expression (*p* = 0.00063; Figure 2B).

### Ovarian Cancer Tissue Microarray Analysis Corroborates Bioinformatics Data

**Figure 3:**
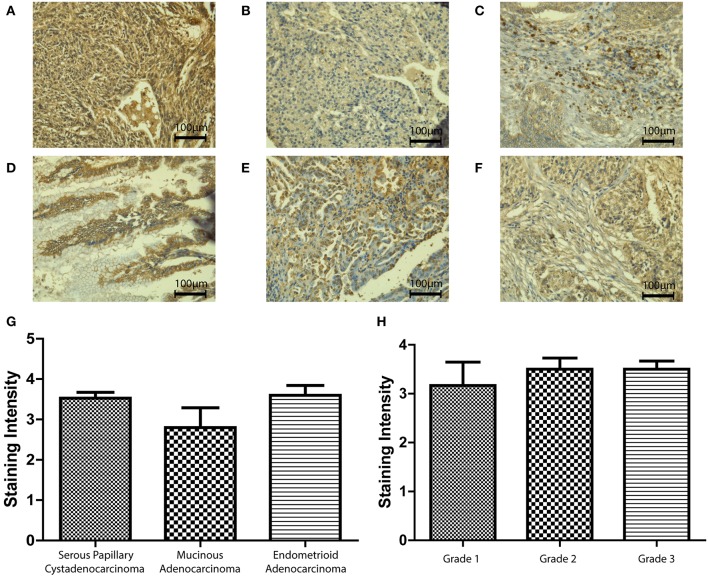
Immunohistochemistry for SP-D expression in different pathologies of ovarian tissue array clinical samples (40x magnification): serous papillary cystadenocarcinoma **(A)**, mucinous adenocarcinoma **(B)** and endometrioid adenocarcinoma **(C)**. No significant change was seen between different types of OC (*p* = 0.2235; **G**). Immunohistochemistry for SP-D expression in different grades of ovarian tissue array clinical samples: mucinous cystadenocarcinoma grade 1 **(D)**, serous papillary cystadenocarcinoma grade 2 **(E)** and serous papillary cystadenocarcinoma grade 3 **(F)**. No significant change was seen between different grades of disease (*p* = 0.7055, **H**).

We then used an ovarian cancer tissue microarray to perform immunohistochemistry in a number of clinical samples in order to validate the *in-silico* data and identify any changes in protein expression with respect to type, grade, or stage of the disease. SP-D was expressed in serous papillary cystadenocarcinoma, mucinous adenocarcinoma and endometrioid adenocarcinoma (Figures 3A–C). Scoring of immunostaining did not reveal any apparent differences in the SP-D expression (Figure 3G), thus corroborating the gene expression Oncomine data. We then measured SP-D expression in clinical samples of different grades: Grade 1 (*n* = *6*), Grade 2 (*n* = *14*) and Grade 3 (*n* = *28*) (Figures 3D–F). No apparent differences amongst grades were noted (Figure 3H).

**Figure 4:**
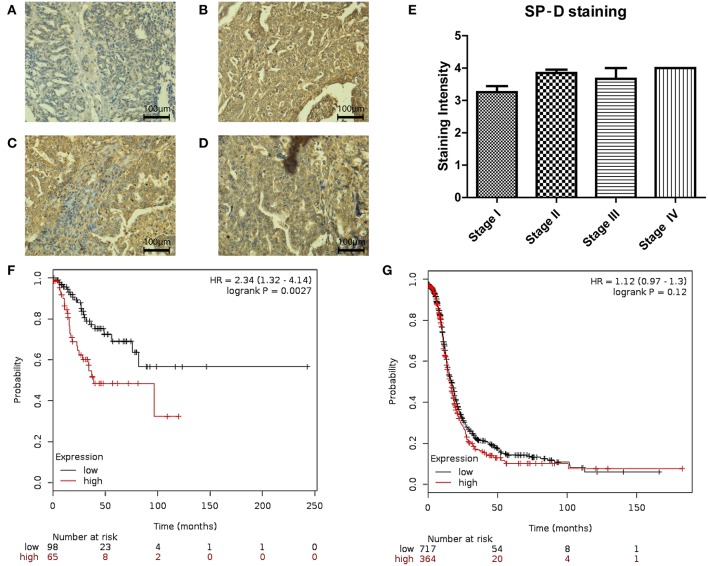
Immunohistochemistry for SP-D expression in different stages of ovarian tissue array clinical samples (40 x magnification): serous papillary cystadenocarcinoma stage I **(A)**, serous papillary cystadenocarcinoma stage II **(B)**, serous papillary cystadenocarcinoma stage III **(C)** and serous papillary cystadenocarcinoma stage IV **(D)**. A nearly significant up-regulation of SP-D was observed in stage II OC compared to stage I OC (*p* = 0.053; **E**). Stratification of PFS in Stages I and II **(F)** against stages III and IV **(G)**. There was a significant survival improvement in low SP-D cohort compared to patients with high SP-D expression only in Stages I and II (*p* = 0.0027, and *p* = 0.12, respectively).

We also measured SP-D protein expression in different stages (I-IV) of ovarian cancer (*n* = 48; Figures 4A–D). No differences were noted between stages III and IV. However, when Stages I and II were compared, SP-D was over-expressed in Stage II compared to Stage I (*p* = 0.053; Figure 4E). When we stratified, PFS in Stages I and II (Figure 4F; *n* = 163) against stages III and IV (Figure 4G; *n* = 1081), there was a significant survival improvement in low SP-D cohort compared to patients with high SP-D expression only in Stages I and II. These results are consistent with Mangogna et al who reported correlation between SP-D expression in ovarian cancer tissues and poor survival prognosis (18).

### SP-D Is Expressed in Circulating Tumor Cells (CTCs) From Ovarian Cancer Patients

**Figure 5:**
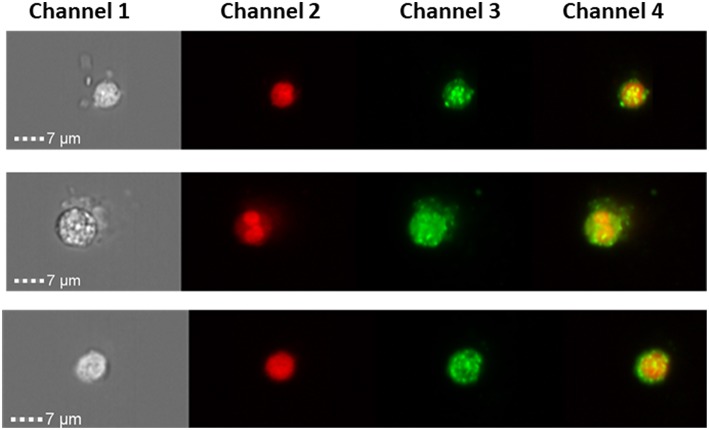
ImageStream analysis showing expression SP-D in circulating tumor cells of 3 ovarian cancer patients, magnification x 40. Channel 1 shows the brightfield pictures of cells, channel 2 shows the nucleus stained with DRAQ5 (red), channel 3 shows SP-D expression using an Alexa Fluor® secondary antibody (green) and channel 4 is the merged image of channels 1 and 2. SP-D is localized in the cytoplasm, with strong fluorescence intensity.

Liquid biopsies offer a promising alternative to tissue samples, providing a non-invasive diagnostic approach or serial monitoring of the disease evolution. Here, we measured SP-D protein expression in CTCs from three Stage III ovarian cancer patients (Figure 5) using a novel imaging flow-cytometry (ImageStream). CTCs were selected based on being positive for cytokeratin markers, WT-1 and negative for CD45 (a white blood cell marker). CTCs from all three patients were positive for SP-D (Figure 5).

### rfhSP-D Compromises Migratory Capacity of SKOV3 Ovarian Cancer Cells *in vitro*

In view of the abundance of SP-D in ovarian cancer tissues, we wished to investigate how SP-D can affect ovarian cancer cells *in vitro*. We used a well-established rfhSP-D, which has been shown to induce apopotosis in pancreatic cell lines, to treat cultured SKOV3 cells.

**Figure 6:**
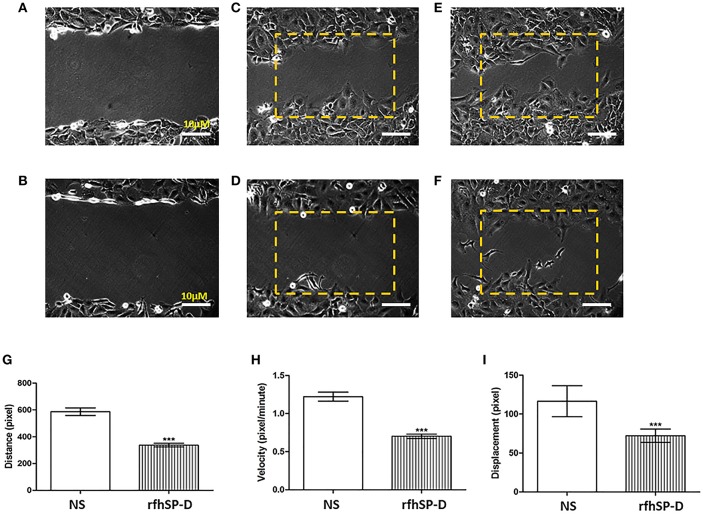
Wound healing assay. The artificial wound created on the cell surface in untreated cells **(A)** and rh-SPD treated cells **(B)** at 0 h. Untreated cells **(C)** or treated cells with rfhSP-D **(D)** did not appear to close the gap after 12 h. At 24 h untreated cells managed to close the gap **(E)** whereas rfhSP-D treated cells did so after 30 h **(F)**. rfhSP-D treated SKOV3 cells have their distance **(G)**, velocity **(H)**, and displacement **(I)** reduced, compared to control SKOV3 cells. 25 cells were counted for each experiment. Comparisons ±SEM between untreated (NS) and treated SKOV3 cells with rfhSP-D at 10 μg/ml (***p < 0.001).

Wound healing assay was used to have a visual assessment of cell proliferative and migratory capacity spatially *in vitro*. Using a fine pipette tip, an artificial wound was created on a confluent cell surface followed by treatment with 10 μg/ml of rfhSP-D. Images acquired from the wound healing assay over 48 h showed that untreated SKOV3 cells closed the gap at 24 h, whereas SP-D treated cells closed the artificially-created gap after 34 h (Figures 6A–F). Therefore, there was a marked inhibition of growth in the treated cells.

We expanded on these observations by locating an area with approximately 25–30 cells where images were captured every 5 min for up to 12 h. Using ImageJ, the coordinates for 25 cells were acquired. Using Pythagora's theorem for each point for each of the cells, the distance, velocity and displacement for all 25 cells were calculated. Cells treated with rfhSP-D moved significantly less and slower compared to untreated cells; there was a significant decrease in distance (Figure 6G), velocity (Figure 6H) and displacement (Figure 6I) of rfhSP-D treated SKOV3 cells compared to untreated cells.

### rfhSP-D Induces Apoptosis in SKOV3 Ovarian Cancer Cells

**Figure 7:**
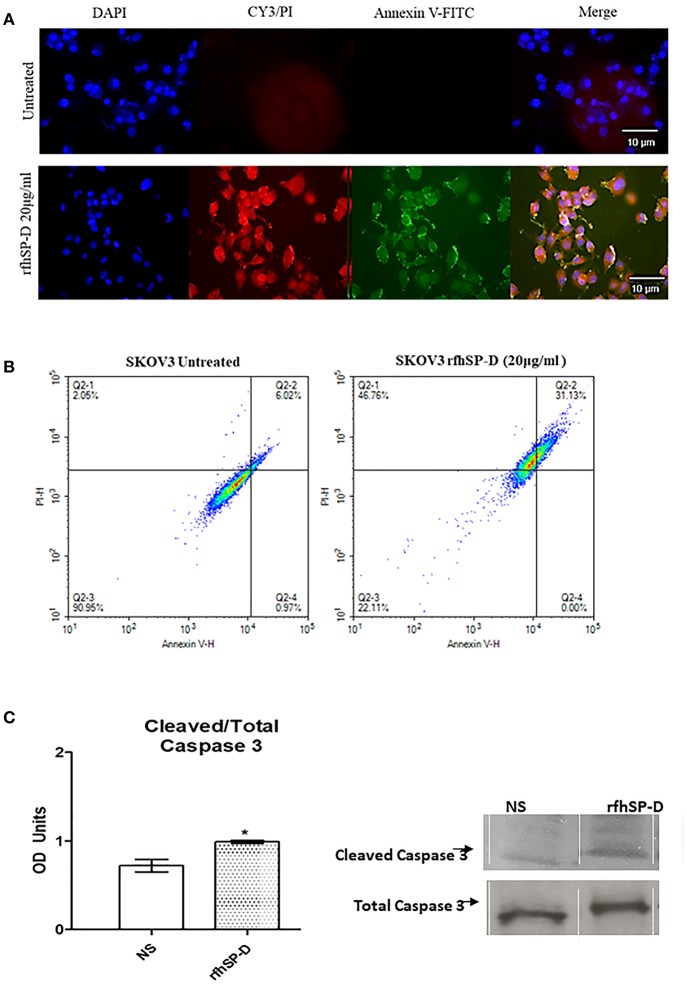
Fluorescence microscopy to analyze apoptosis in SKOV3 cells treated with rfhSP-D for 48 h. Apoptosis was analyzed using an annexin V/propidium iodide (PI) staining kit **(A)**. The cell membrane was positively stained for annexin V and the DNA staining is visible in the treated cells indicating that the cells underwent apoptosis and PI was taken in which stained the DNA of apoptotic cells. No such staining was seen in the untreated cells. The nucleus was stained with Hoechst for both treated and untreated cells **(A)**. Quantitative analysis of apoptosis using Flow Cytometer **(B)**. SKOV3 cells were treated with rfhSP-D 48 h and apoptosis was analyzed using annexin V with PI kit. Cells were acquired and plotted for both annexin V/FITC, which showed a shift in the fluorescence intensity of in treated cells **(B)**. Effects of rfhSP-D on caspase 3 **(C)**. Scanning densitometry of protein expression of cleaved over total caspase 3 in SKOV3 cells treated with rfhSP-D ±SD at 48 h which were acquired from the western blotting analysis **(C)**. Cleaved Caspase 3 over total ratio was significantly increased after 48 h 20 μg/ml. *p < 0.05.

Fluorescence microscopy analysis of rfhSP-D treated SKOV3 cells revealed a positive staining for cell membrane integrity marker, Annexin V (conjugated to FITC), and disoriented cell membrane morphology was observed. Thus, PI positive staining was seen in rfhSP-D treated cells compared to un-treated controls (Figure 7A), suggesting induction of apoptosis by the protein.

The quantitative as well as qualitative analyses of apoptosis induction in SKOV3 cells were performed using immunofluorescence microscopy and flow cytometry. A significant proportion of SKOV3 cells following rfhSP-D treatment showed apoptosis at 48, as revealed by FACS analysis. 20 μg/ml of rfhSP-D was effective in inducing the maximum apoptosis in this cell line at 48 h; i.e., ~68%. Approximately 31% cells were FITC and PI positive, suggesting that annexin V/FITC was able to bind to phosphatidylserine (PS) found on the cell surface of cells undergoing apoptosis. However, the percentage of SKOV3 cells stained for PI alone was higher (~46%), suggesting that these cells were either at the late stage of apoptosis or undergoing necrosis. The percentage of viable cells in the untreated sample (90%) was significantly higher as compared to treated sample, suggesting that apoptosis induction was protein-specific, where the integrity of the cell membrane was intact in the untreated cells, and hence, the cells were still viable (Figure 7B).

In view of these findings, we sought to investigate the effects of rfhSP-D on the cleavage of caspase-3, a critical mediator of apoptosis. Cleaved caspase-3 compared to total caspase-3 expression was increased after 48 h at 20 μg/ml rfhSP-D treatment (Figure 7C).

**Figure 8:**
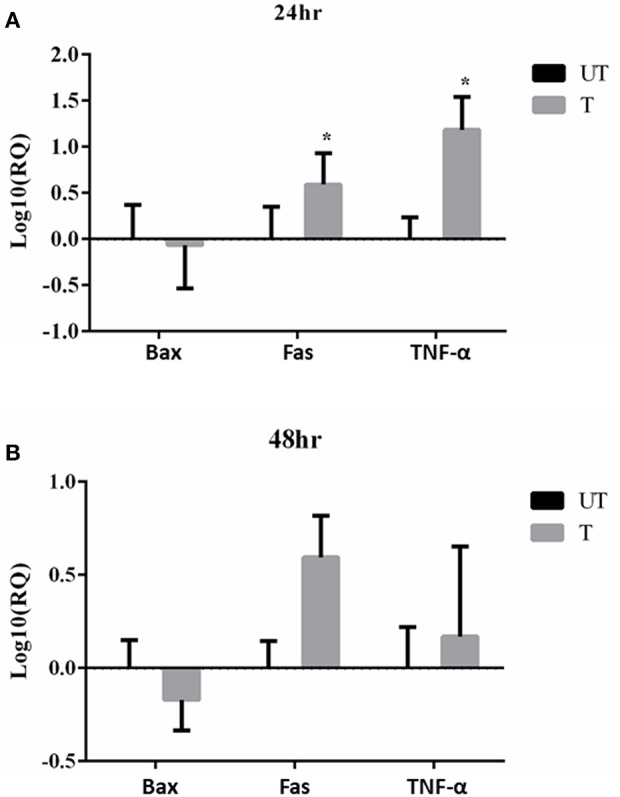
Effects of rfhSP-D treated SKOV3 cells on Bax, Fas and TNF-α at 24 h **(A)** and 48 h **(B)**. Fas and TNF-α are significantly up-regulated at 24 h of treatment compared to controls *P < 0.05.

Moreover, changes in the expression of pro-apoptotic genes, Bax, Fas and TNF-α following treatment with rfhSP-D (20 μg/ml) at 24 and 48 h were assessed. We chose to include TNF-α since it is a key mediator of the apoptotic pathway and has been shown to modulate Fas expression. Bax was unaffected following the treatment with rfhSP-D in SKOV3 cells at both time-points (Figures 8A,B), suggesting that the intrinsic apoptotic pathway may not be directly involved. Fas and TNF-α were significantly upregulated following treatment with rfhSP-D at 24 h compared to controls (Figure 8A). At 48 h, there was no apparent changes in the mRNA expression of TNF-α, but a clear trend in the over-expression of Fas was noted (Figure 8B).

### Treatment of SKOV3 Cells With rfhSP-D Compromises mTOR Signaling

**Figure 9:**
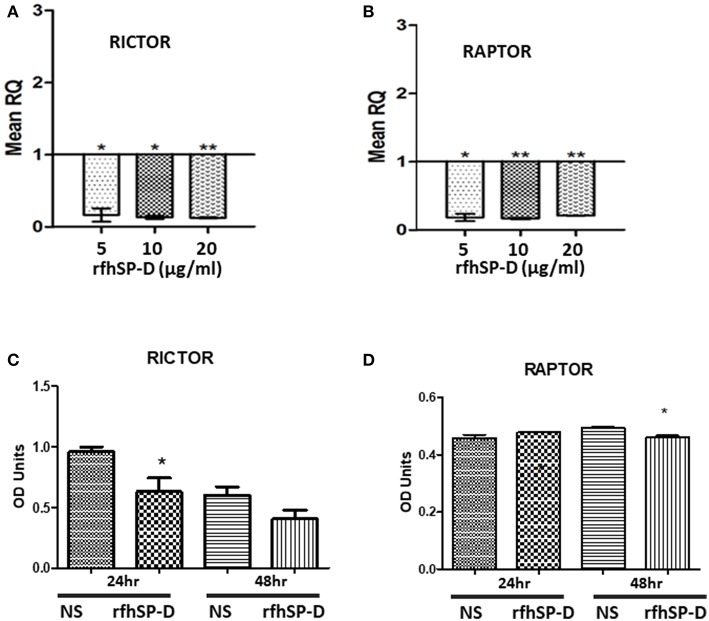
Relative quantification of Rictor **(A)** and Raptor **(B)** mRNA expression in SKOV3 cells treated with 5, 10 and 20 μg/ml of rfhSPD after 12 h (*p < 0.05, **p < 0.01 compared to basal levels set at 1.0). Western blotting for Rictor **(C)** and Raptor **(D)** showed a downregulation at protein level at 24 and 48 h, respectively compared to controls; *P < 0.05.

The mechanistic Target of Rapamycin (mTOR) has been linked with the pathogenesis of ovarian cancer, especially with its progression (33). Treatment of SKOV3 cells with rfhSP-D (5, 10 and 20 μg/ml) for 12 h did not affect the gene or protein expression of mTOR (data not shown). However, mRNA levels of both key components of mTORC1 and mTORC2 complexes, namely Raptor and Rictor, were significantly down-regulated 12 h post-treatment (Figures 9A,B). Rictor downregulation was mirrored at protein level at 24 h as assessed by Western blotting (Figure 9C), whereas a reduction in Raptor expression was evident at 48 h post rfhSP-D (20 μg/ml) treatment (Figure 9D).

**Figure 10:**
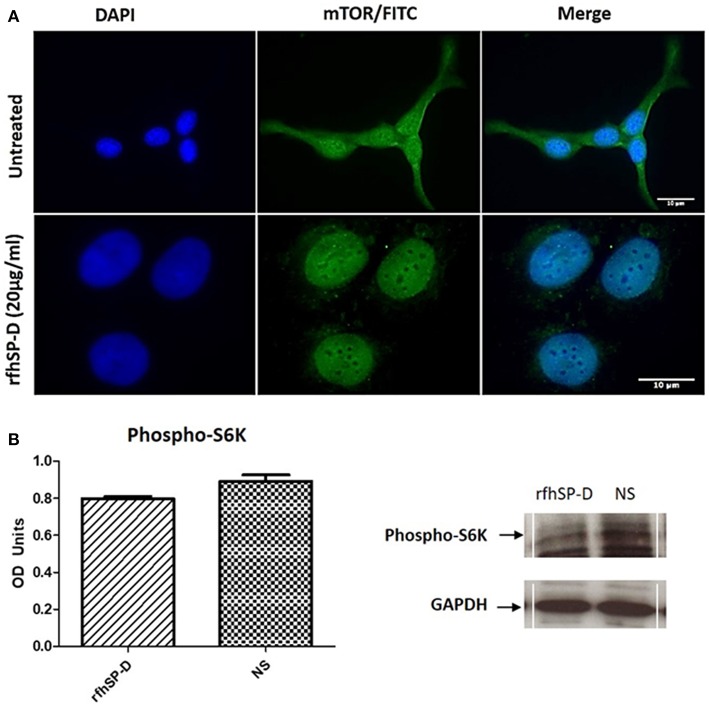
Assessment of mTOR localization in SKOV3 cells. **(A)** rfhSP-D treatment downregulated the survival pathway, mTOR. Fluorescence microscopy analysis following rfhSP-D (20 ug/ml) treatment of SKOV3 cells showed reduced cytoplasmic levels of mTOR following treatment as compared to the untreated. Nuclear accumulation of mTOR was seen in the treated cells. **(B)** Effects of rfhSP-D on phospho (Thr389) p70S6K in SKOV3 cells treated with rfhSP-D (20 μg/ml).

Fluorescence microscopy analysis following rfhSP-D (20 μg/ml) treatment of SKOV3 cells for 12 h showed reduced cytoplasmic levels of mTOR compared to the untreated controls (Figure 10A). Nuclear accumulation of mTOR was seen in the treated cells, indicative of a potential shuttling.

Finally, we assessed the effect of rfhSP-D on mTORC1 activity by measuring the phosphorylation status of S6 Kinase (S6K) since an increase in S6K phosphorylation is a sign of an active mTOR pathway and has been evidenced in late stage ovarian cancer (19). There was a notable (*p* = 0.05) decrease in the phosphorylation level of (Thr389) p70S6K S6K protein following rfhSP-D (20 μg/ml) treatment (Figure 10B).

## Discussion

SP-D, which acts as an anti-microbial and anti-allergic humoral factor at the mucosal surface, has emerged as an innate immune surveillance molecule against cancer. Its transcriptional and protein levels have recently been associated with prognosis in a range of cancers (18). SP-D has been reported to be overexpressed in ovarian cancer patients (serous cystadenocarcinoma) compared to healthy controls (18). Here, we extended our observations by measuring the gene expression levels in different sub-types of ovarian cancer. We provide evidence that SP-D is widely expressed in clear cell adenocarcinomas, endometrioid adenocarcinomas, mucinous adenocarcinomas, and serous adenocarcinoma. We validated these observations by immunohistochemistry using tissue microarrays, where SP-D expression was unchanged in grade or type; however, there was a modest increase in the expression between Stage I to Stage II.

Low SP-D mRNA expression showed a significant improvement in overall survival and progression free survival than higher SP-D expression. A report on non-small cell lung cancers (NSCLC) revealed that an increased expression of SP-D in the lungs of NSCLC patients was correlated with metastasis and poor outcome (34). These data provide evidence for a higher order of complexity in the regulation and function of SP-D in cancer. Another study has recently reported that in lung, gastric, and breast cancers, there is a lower expression of SP-D compared to healthy controls; in lung cancer, the presence of SP-D could be associated with a favorable prognosis. On the contrary, in breast, and ovarian cancers, the presence of SP-D is associated with an unfavorable prognosis (18). It is possible that SP-D acts in a tissue- or cell-specific manner, depending on the composition of the tumor microenvironment. These observations require further investigation using a wider repertoire of cell lines and clinical samples. Nevertheless, SP-D emerges here as a potential biomarker for ovarian cancer.

Thus, we expanded on the clinical observations by measuring its expression in circulating tumor cells (CTCs) of 3 Stage III ovarian cancer patients. An abundant expression was noted using an imaging flow-cytometric approach. This is the first time that SP-D expression is being documented in CTCs. Blood based biomarkers such as “liquid biopsies” are becoming an increasingly attractive area of cancer research due to their non-invasive nature and importance in detecting the stage and the spread of the tumor (35). The detection of CTCs and appropriate biomarkers can, therefore, help not only in diagnosis but also in real-time therapy monitoring and more efficient personalized treatments (36).

We also demonstrate an involvement of SP-D in ovarian cancer and provide a novel insight into the signaling pathways that are involved using an ovarian cancer cell line *in vitro*. When SKOV3 cells were treated with rfhSP-D, there was a decrease, although moderate, in proliferation, suggesting a direct anti-proliferative effect of rfhSP-D. There was also a noticeable decrease in distance, velocity and displacement of SKOV3 cells treated with rfhSP-D, when compared to untreated cells. Thus, rfhSP-D could exert a cytostatic or cytotoxic effect, as evident from rfhSP-D induced Caspase 3 cleavage *in vitro*. Caspase-3 is a critical regulator of apoptosis, as it contributes to the proteolytic cleavage of many key proteins, such as poly adenosine diphosphate-ribose polymerase (PARP) (37). The rfhSP-D induced apoptosis in SKOV3 cells was further corroborated by an increase in annexin V staining, which is used to detect cell death by its ability to bind to PS, a marker of apoptosis when it is on the outer leaflet of the plasma membrane. Collectively, our study is in agreement with recent studies involving a number of malignancies. In a human lung epithelial cancer cell line (A549), SP-D inhibits cell progression and metastasis via inhibition of the EGF pathway through binding to EGFR and compromising the EGFR-EGF complex (12). rfhSP-D can also alter oxidative stress and high-mobility group A factor 1 (HMGA1) expression leading to induction of the p53 apoptotic pathway in an eosinophil leukemic cell line (7, 8). In a more recent study, rfhSP-D induced apoptosis in pancreatic cancer cell lines via Fas-mediated pathway, followed by cleavage of caspases 8 and 3 (16).

In this study, we demonstrate that rfhSP-D exerts its anti-proliferative effects by compromising mTOR signaling and activating the pro-apoptotic genes, Fas and TNF-α, but not Bax. Fas belongs to TNF superfamily that can activate caspase cascade, which can lead to cleavage of caspase 3 as the terminal molecular event during apoptosis (38–40). TNF-α, another TNF superfamily member, exerts its effects via TNFR2 to increase the susceptibility of the target cells to Fas-mediated death (16, 41). With regards to Bax, this is not the first time that a modest gene expression is noted. When FSTL1 and RAD51AP1 genes were silenced in ovarian cancer cells, a decrease in proliferation was noted while the Bax gene expression remained unchanged (24, 42).

To this date, little is known about an association between SP-D and mTOR. The mTOR pathway has been shown as an important regulatory epicenter for cell growth, metabolism, autophagy, gene expression and protein synthesis (43). mTOR acts through two complexes, mTORC1 and mTORC2, and Raptor and Rictor are key components of these two complexes, respectively (4, 44). We, therefore, decided to examine the effects of rfhSP-D on the mTOR pathway. When we treated SKOV3 cells with rfhSP-D and measured the expression of key components of the mTOR pathway, the mRNA and protein levels of Rictor and Raptor were down-regulated compared to untreated controls, with a concomitant decrease in the phosphorylation of S6K (Ser).

Interestingly, although the mTOR protein expression remained largely unaltered following rfhSP-D treatment, its translocation from the cytoplasm to the nucleus was noted. mTOR primarily resides within the cytoplasm (45), but nuclear localization has also been documented in rhabdomyosarcomas, human fibroblasts and colon carcinoma cells (46). In a study using multiple myeloma cell lines, it was reported that treatment with pomalidomide (IMID®), an immunomodulatory FDA-approved drug for the treatment of multiple myeloma, increased nuclear mTOR and p-mTOR expression levels in the nucleus with a concomitant decrease of the cytoplasmic fractions (47). This shuttling correlated with a decrease in cell viability in this study. Moreover, it has been shown that the mTORC2 complex components, Rictor and Sin1, are dephosphorylated and dynamically distributed between the cytoplasm and the nucleus upon long-term treatment with the mTOR-inhibitor rapamycin (48). Thus, rfhSP-D can compromise mTOR signaling via downregulation of Rictor and Raptor, shuttling of mTOR leading to dysfunction of the downstream signaling cascade (i.e., dephosphorylation of S6K), and subsequent inhibition of cell proliferation.

In conclusion, rfhSP-D treatment of SKOV3 cells downregulates Rictor and Raptor and upregulates pro-apoptotic factors such as TNF-α and Fas to activate caspase 3 cascade to induce apoptosis *in vitro*. New therapeutic strategies involving immune molecules such as rfhSP-D that target pro-apoptotic genes and the mTOR pathway merit further investigation. Finally, the emergence of SP-D as a potential biomarker needs to be verified in a large cohort of ovarian cancer patients. However, the prognostic value of SP-D in ovarian cancer, where high levels correlate with poor survival, together with the *in vitro* effects of rfhSP-D, is quite intriguing. One possibility is that the SP-D protein in ovarian cancer tissues is non-functional as we have recently observed in a triple-positive breast cancer cell line that secretes a non-functional variant of SP-D although the same cell line remains susceptible to apoptosis induction by rfhSP-D (Murugaiah et al., unpublished data). In case the ovarian tissue, SP-D is functionally active, it is possible it binds to components of tumor microenvironment (e.g., hyaluronic acid). This interaction is capable of negating the protective effect of SP-D *in vivo*. Further studies are required to delineate the relationship between SP-D, its ligand in the tumor microenviroment and SP-D receptors on the primary ovarian cancer cells.

## Data Availability

Publicly available datasets were analyzed in this study. This data can be found here: www.oncomine.org.

## Ethics Statement

Patients included in this study were enrolled in the prospective CICATRIx clinical study which collects blood samples for exploratory biomarkers from patients with advanced cancer attending Mount Vernon Cancer Centre (East and North Hertfordshire NHS Trust). All patients provided written informed consent for participation in the study and for use of their donated tissue and blood specimens. The CICATRIx study was approved by the West Midlands – South Birmingham Ethics Committee (reference 16/WM/0196).

## Author Contributions

JK and VM carried out key experiments. GS, AK, JJ, and IS carried out supportive and validation experiments. FA, JC, and MH provided crucial reagents. UK and EK designed various experiments and wrote the manuscript.

### Conflict of Interest Statement

The authors declare that the research was conducted in the absence of any commercial or financial relationships that could be construed as a potential conflict of interest.
